# Optimizing Corticosteroid Sinonasal Irrigation Outcomes Through 3D Printing: A Randomized Pilot Clinical Trial

**DOI:** 10.1002/oto2.70036

**Published:** 2024-10-16

**Authors:** Zachary T. Root, Thomas J. Lepley, Kanghyun Kim, Aspen R. Schneller, Songzhu Zhao, Raymond Wen, Veronica L. Formanek, Sarah M. Sussman, Joseph S. Lee, Ahmad Odeh, Lai Wei, Kathleen M. Kelly, Bradley A. Otto, Kai Zhao

**Affiliations:** ^1^ Department of Otolaryngology–Head and Neck Surgery The Ohio State University Wexner Medical Center Columbus Ohio USA; ^2^ Department of Biomedical Informatics and Center for Biostatistics The Ohio State University Wexner Medical Center Columbus Ohio USA

**Keywords:** chronic rhinosinusitis, FESS, nasal drug delivery, steroid, topical medication

## Abstract

**Objective:**

Topical corticosteroid irrigation plays critical role in the management of chronic rhinosinusitis (CRS). Yet, its efficacy can be highly variable. We sought to determine if personalized, 3‐dimensional (3D)‐printed nasal models can optimize head positioning and irrigation parameters, therefore improving patient outcomes.

**Study Design:**

Randomized, single‐blinded clinical trial.

**Setting:**

Tertiary medical center from November 2021 to July 2023.

**Methods:**

Sixty‐two patients with CRS were randomized into either control (CG), backfill (BG), or model (MG) groups; daily 2 mg mometasone irrigations were then performed for 2 months with either standard head‐forward and natural side‐tilt position (CG), a head tilt of 90° to the side with fluid entering the lower nostril (BG), or in an optimized position as determined by a patient‐specific 3D printed irrigation model (MG), respectively.

**Results:**

A total of 36 patients completed the trial (CG: N = 14/23; BG N = 11/23, MG: N = 11/16). Significant posttreatment improvement in Lund‐Mackay (LM) scoring was only observed in the MG (−3.73, 95% confidence interval = −5.71, −1.75; *P* < .001). Patient‐reported outcome measures (Nasal Obstruction Symptom Evaluation, Sinonasal Outcome Test‐22, and Visual Analog Scale of nasal congestion) improved significantly among all groups. Optimal model penetration scores significantly correlated to posttreatment MG LM score (Spearman's *r* = 0.65, *P* < .05). Among all groups, patients with prior endoscopic sinus surgery (ESS) (n = 19) had objectively less opacification at baseline; however, experienced the same degree of opacification reduction and symptom reduction as those without prior ESS (n = 17).

**Conclusion:**

The use of 3D printing to personalize head positioning may significantly improve objective corticosteroid irrigation outcomes. Mometasone irrigation may have similar subjective and objective effects on patients regardless of prior surgical history.

**Level of Evidence:**

Level 1 prospective, randomized, single‐blinded clinical trial NCT06118554.

Chronic rhinosinusitis (CRS) is a common medical condition that impacts the quality of life for an estimated 10% to 13% of adults.^1^ Reported symptoms often include nasal obstruction, facial pain/pressure, rhinorrhea, postnasal drip, and hyposmia. CRS can have a negative impact on quality of life beyond rhinologic symptoms and can lead to chronic ear symptoms, poor sleep quality, and psychological disturbances, among others.^2^

Topical intranasal steroids are among the hallmarks of CRS management. While these medications can be applied in the nose using a variety of methods, including sprays, atomizers, and nebulizers, high‐volume irrigation has been shown to be more effective in distributing medication to the paranasal sinuses.^3^, ^4^, ^5^, ^6^, ^7^, ^8^ Clinical trials have shown that the use of corticosteroid delivered by irrigation is superior to other methods in the setting of diffuse or patchy CRS in postsurgery patients.^5^ Furthermore, multicenter surveys have shown that patients appreciate the ability to control the pressure, temperature, and volume of their irrigation, which may lead to better compliance.^9^ However, due to the intricate and variable anatomy of the human nasal airway, the efficacy of topical irrigation to reach targeted sinuses is inconsistent and difficult to predict. Previous studies have shown that nasal irrigation may not reliably penetrate all sinuses,^10^ and the effectiveness depends on specific sinuses, head positions, injection angle, flow rates, and individual anatomy.^11^, ^12^ As a result, there may not be a single method that could be optimal for the general population.

The primary method of investigating nasal irrigation has been limited to cadaver studies or the use of colored dyes under endoscopic visualization.^10^, ^11^ Other studies have used irrigation with iodinated contrast followed by computed tomography (CT) scans to determine which sinuses collect contrast material,^4^, ^13^ or technetium 99 m sulfur colloid and fluorescein^14^ labeled irrigation to determine the distribution of sinus irrigation. These are labor‐intensive techniques that may present a risk to the patient and are difficult to scale up to a larger patient population. While these studies can provide some insight into anatomy, they typically use a small sample size and cannot account for all anatomic variations, making it difficult to generalize conclusions that can be translated to patient care. Computational fluid dynamics (CFD) has also been used to simulate sinonasal irrigation and may prove useful in determining the optimal degree of surgery or a personalized irrigation strategy to allow for targeted irrigant penetration into the sinuses. CFD has some advantages over other methods: it is less labor‐intensive as it requires only a CT scan; it captures the complete flow path of the irrigation as it is pushed from the tip of the irrigation delivery device through the sinonasal cavities; it is patient‐specific and imparts no risk or discomfort to the patient.^15^, ^16^, ^17^ However, a major drawback of CFD is the time it takes to complete—even with parallel processing through multicore central processing units or computer clusters.

Recently, a novel alternative to these approaches has emerged in the form of additive manufacturing, where CT scans obtained during routine clinical workup can be printed into anatomically accurate 3‐dimensional (3D) models. These models have been used to observe differences in irrigant sinus penetration at different head positions and to identify which positions are optimal for each sinus.^18^, ^19^, ^20^ However, no study correlates these 3D printing results to patient care and clinical outcomes in a large cohort.

Here, we performed the first randomized clinical trial using 3D‐printed sinonasal models to determine if developing a personalized nasal irrigation method is more effective in managing CRS than using the traditional irrigation method or a recently studied “backfill” method. Additionally, we sought to understand whether the use of a 3D model as a visual teaching aid had an impact on patient compliance or if past surgical history influenced treatment outcomes.

## Methods

### Subjects

**Figure 1:**
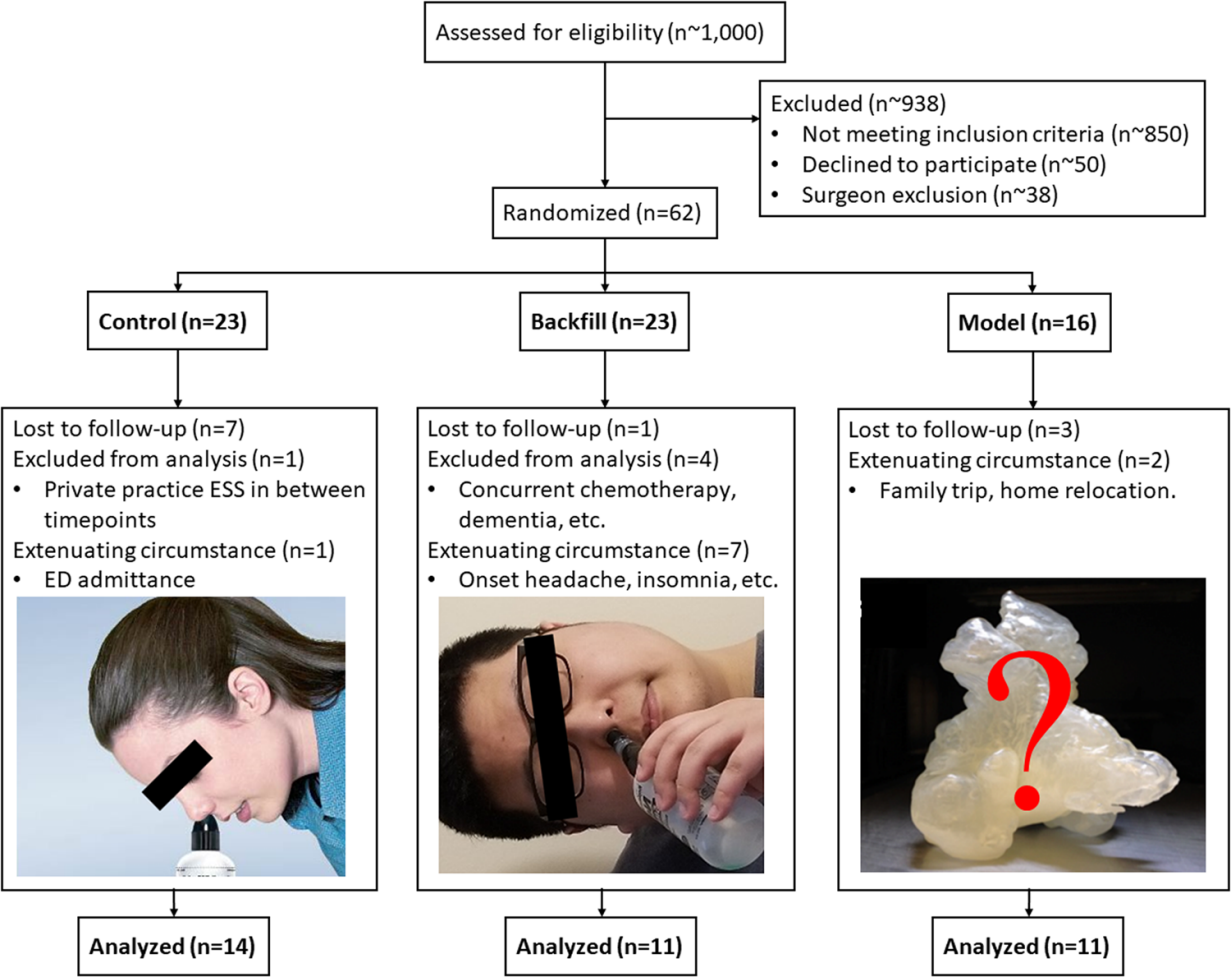
Consolidated Standards of Reporting Trials flow diagram demonstrating patient's randomization into the 3 irrigation treatment groups. Reasons for patient dropout are listed.

Sixty‐two patients (32 males, 30 females, aged 53.06 ± 15.70 years) with CRS were enrolled and randomized to either the control (CG), backfill (BG), or model (MG) groups (Figure 1, Table 1). The clinical diagnosis of CRS was determined by 2 fellowship‐trained rhinologists and based on international practice guidelines.^21^, ^22^ Based on previous studies,^23^ a sample size of 20 subjects per group would provide an 80% power to detect an effect size of 0.81 standard deviations in Sinonasal Outcome Test (SNOT)‐22 score changes from baseline to follow‐up between the CG and MG with an estimated 20 ± 10 drop in SNOT‐22 score among the CG and 32 ± 16 drop among the MG (with *α* = .05). Two clinical research coordinators enrolled and assigned patients to one of the three groups using a random number generator. Patients having baseline clinical CT scans with high (>100) slice counts were preferentially assigned to the MG due to the high spatial resolution needed to print anatomically accurate 3D models. The slice count may reflect a biased selection of patients, though it may also reflect variations of imaging facilities that patients may choose depending on their schedules, availability of appointments, and location preferences. A majority of patients received their CT scan at our university's flagship rhinology clinic where the research team has direct access to the CT scanner. However, for patients receiving their CT scan at a separate location with a less modern scanner, the scans had to be obtained from the Picture Archiving and Communication System where the slice count stored was reduced. Group assignments were blinded to patient's medical history or symptom severity. Patients with significant nasal polyps completely blocking all ostia (as determined by a clinician) were excluded upfront due to the unlikeness of irrigation penetration regardless of irrigation paradigm optimization. Patients with allergic rhinitis, cystic fibrosis, granulomatosis with polyangiitis or other connective tissue disorder, and current pregnancy were also excluded. The study was single‐blinded as the MG patients unavoidably knew of their experimental group assignment due to the provided 3D‐printed nasal model. However, patients in BG and CG were not told the existence of other groups. Clinical providers were blinded to the patients' assigned group when assessing clinical outcomes and Lund‐Mackay score, and the principal investigator and 3D printing team were blinded to the patients' clinical outcomes.

**Table 1:** Demographic Characteristics Among the Groups

Characteristic	Control (n = 14)	Backfill (n = 11)	Model (n = 11)	*P* value
Sex No. (%)				.099
Male	5 (35.7%)	8 (72.7%)	8 (72.7%)	
Female	9 (64.3%)	3 (27.3%)	3 (27.3%)	
Age, mean (SD), y	58.7 (10.5)	50.7 (16.7)	47.8 (18.4)	.188
Race and ethnicity No. (%)				.237
White	12 (85.7%)	9 (81.8%)	6 (54.5%)	
Black	1 (7.1%)	2 (18.2%)	3 (27.3%)	
Asian	1 (7.1%)	0	1 (9.1%)	
Hispanic	0	0	1 (9.1%)	
Prior surgical history				.148
No	5 (35.7%)	4 (36.4%)	8 (72.7%)	
Yes	9 (64.3%)	7 (63.6%)	3 (27.3%)	
CRSwNP				.214
Yes	4 (28.6%)	7 (63.6%)	5 (45.5%)	
No	10 (71.4%)	4 (36.4%)	6 (54.5%)	
Lund‐Mackay, mean (SD)	Missing = 3	Missing = 6	Missing = 0	.006
	7.1 (3.1)	13.7 (5.2)	11.3 (4.6)	
NOSE, mean (SD)	58.6 (27.1)	59.1 (17.3)	56.4 (19.1)	.953
SNOT‐22, mean (SD)	47.3 (29)	39.9 (16.1)	42.9 (18.8)	.719
VAS, mean (SD)	4.8 (2.7)	5.1 (2.1)	4.6 (2.2)	.880

Symptom scores are scores at baseline. *P* values were produced by the following methods for the following characteristics: age, ANOVA; baseline scores, Kruska‐Wallis test; categorical variables and Fisher's exact test.

Abbreviations: ANOVA, analysis of variance; CRSwNP, chronic rhinosinusitis with nasal polyps; No., number, NOSE, Nasal Obstruction Symptom Evaluation scale; SD, standard deviation; SNOT‐22: Sinonasal Outcome Test‐22; VAS, visual analog scale of nasal obstruction.

The study was approved by the Ohio State Biomedical Sciences Institutional Review Board (approval number: 2020H0068) and is registered on clinicaltrials.gov (NCT06118554). All subjects signed consent and authorization forms for participation and inclusion of their data in this study. Data was collected at the Ohio State University Wexner Medical Center Eye and Ear Institute from November 2021 to July 2023.

### Study Protocol

All patients were prescribed an 8‐week course of daily 2 mg of mometasone irrigation (Advanced Rx Compounding Pharmacy, Fort Washington, PA) using 240 mL Sinus Rinse bottles (NeilMed Pharmaceuticals Inc, Santa Rosa, CA). The CG is irrigated in the standard Food and Drug Administration (FDA) consumer‐recommended position,^21^ defined as leaning forward with a natural ear‐to‐shoulder head tilt (Figure 1). BG subjects were irrigated with a head tilt of 90° ear‐to‐shoulder and used the nostril closest to the ground. Previous computational and 3D printing studies indicate that pushing irrigant against gravity through the lower nostril can allow irrigant to pool around the ostium for improved penetration into paranasal sinuses,^20^, ^24^ although this has not been tested in clinical trials. Finally, the MG was irrigated in an optimal position based on their 3D nasal replica. This patient‐specific position was communicated to each MG patient with clear instructions during an in‐person training session. All patients were required to record their daily compliance if they changed head position, and what head position they changed to during the course of the treatment. Prior sinus surgery and medication history were documented.

During the clinical visit at baseline and post the 8‐week irrigation, all participants completed primary outcome measures: Nasal Obstruction Symptom Evaluation (NOSE) score, SNOT‐22, and visual analog scale (VAS) of nasal congestion (0: *being completely clear*, 10: *being completely blocked*). The severity of inflammation and mucosal thickening was scored blindly and independently by 3 scorers on pre‐ and posttreatment CT scans using the LM CT staging score.^25^ The assessments were found to be consistent between the independent scorers with significant correlations (*r *= ∼.93‐.96, *P* < .05). Secondary outcome variables included past surgical history and sinus penetration scores obtained while experimenting with the nasal models.

### 3D Modeling

For the MG, each patient's CT scan was converted into a sinonasal model with 3D patient‐specific features using computer‐aided design software as previously described.^19^ First, the interface between the nasal mucosa and the air was delineated on the CT scans using image processing software (Amira, Visualization Sciences Group). Then, a ∼3 mm thick wall was created to enclose the nasal air space. The wall's thickness was chosen to structurally support the printed model and to provide a clear view. The wall was specially designed not to encroach into the nasal or sinus airspace nor disrupt the thin bony walls at various delicate regions. The 3D nasal digital model was then 3D printed with a Formlabs Form 2 Stereolithography 3D printer. To determine the optimal strategy for targeted sinuses, irrigations were performed on the 3D models at various head positions, for example, 90° side tilt, 45° side tilt, 45° side and 45° forward tilt, 45° forward tilt, 90° forward tilt. Each position was performed with fluid entering the upper nostril (“conventional”) and the nostril lowest to the ground (“backfill”) to determine the optimal irrigation paradigm. Fluid penetration to the frontal, ethmoid, maxillary, and sphenoid sinuses was graded immediately prior to ceasing the bottle squeeze on a scale of 0 (*none*), 1 (*partial, less than 50%*), 2 (*greater than 50%*), and 3 (*completely filled*). Rubber/silicon seals were attached to the model's nostrils to create a water‐tight seal and food color dye was added for better visualization of fluid penetration.

### Statistical Analysis

The Department of Biostatics was consulted to use a linear mixed model, rather than a simple descriptive comparison method (ie, Student's *t* test), to examine the changes of outcome measures from baseline among 3 treatment groups. A linear mixed model can control for multiple potential confounding factors, including a difference in baseline scores, age, gender, surgical history, and missing data. Analysis of variance was used to compare pre‐treatment variables among the 3 treatment groups. Linear mixed models with random intercept were used to examine the change in outcome measures (post vs pre) by different groups while controlling for prior surgical history (outcome = group + timepoint + group × timepoint + prior surgical history). To analyze the changes of outcome measures from baseline by prior surgical history while controlling for the treatment group, we utilized a mixed model with the following structure: outcome = prior surgical history + timepoint + prior surgical history × timepoint + group. Statistical significance was defined as *P* < .05. Analyses were performed using SAS 9.4 (SAS Institute Inc).

## Results

Out of 62 enrolled patients, 11 patients were lost to follow‐up (CG = 7, BG = 1, MD = 3); 10 were removed due to extenuating circumstances (eg, emergency department admittance, onset headache, insomnia, family trip, home relocation, etc); 5 patients completed the study but were excluded from the analysis due to confounding factors during the treatment process (e.g. unadvised sinus surgery at a private practice, the onset of chemotherapy, dementia, etc). A total of 36 patients were included in the final analysis, with 14 CG, 11 BG, and 11 MG patients. More voluntary drop‐outs occurred among CG patients (7/23 = 30.4%) as compared to the MG (3/16 = 18.8%) and BG (1/23 = 4.35%).

**Figure 2:**
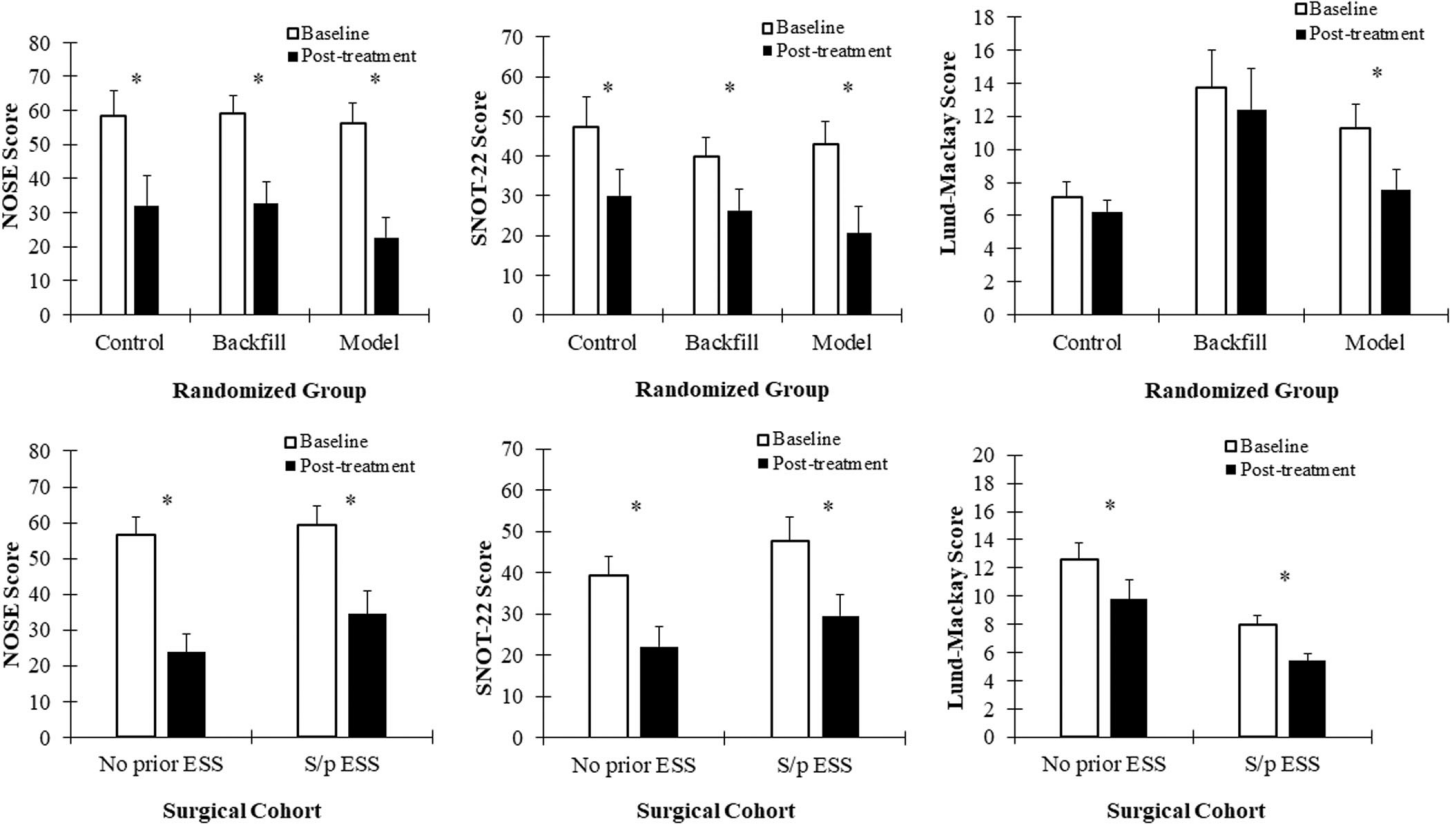
Plots of PROM and Lund‐Mackay score improvement after treatment among the randomized groups, as well as among the cohorts with or without prior surgery. ESS, endoscopic sinus surgery; NOSE, Nasal Obstruction Symptom Evaluation scale; PROM, patient‐reported outcome measure; SNOT‐22, Sinonasal Outcome Test‐22. *indicates statistical significance of *p* < 0.05.﻿﻿﻿﻿

**Figure 3:**
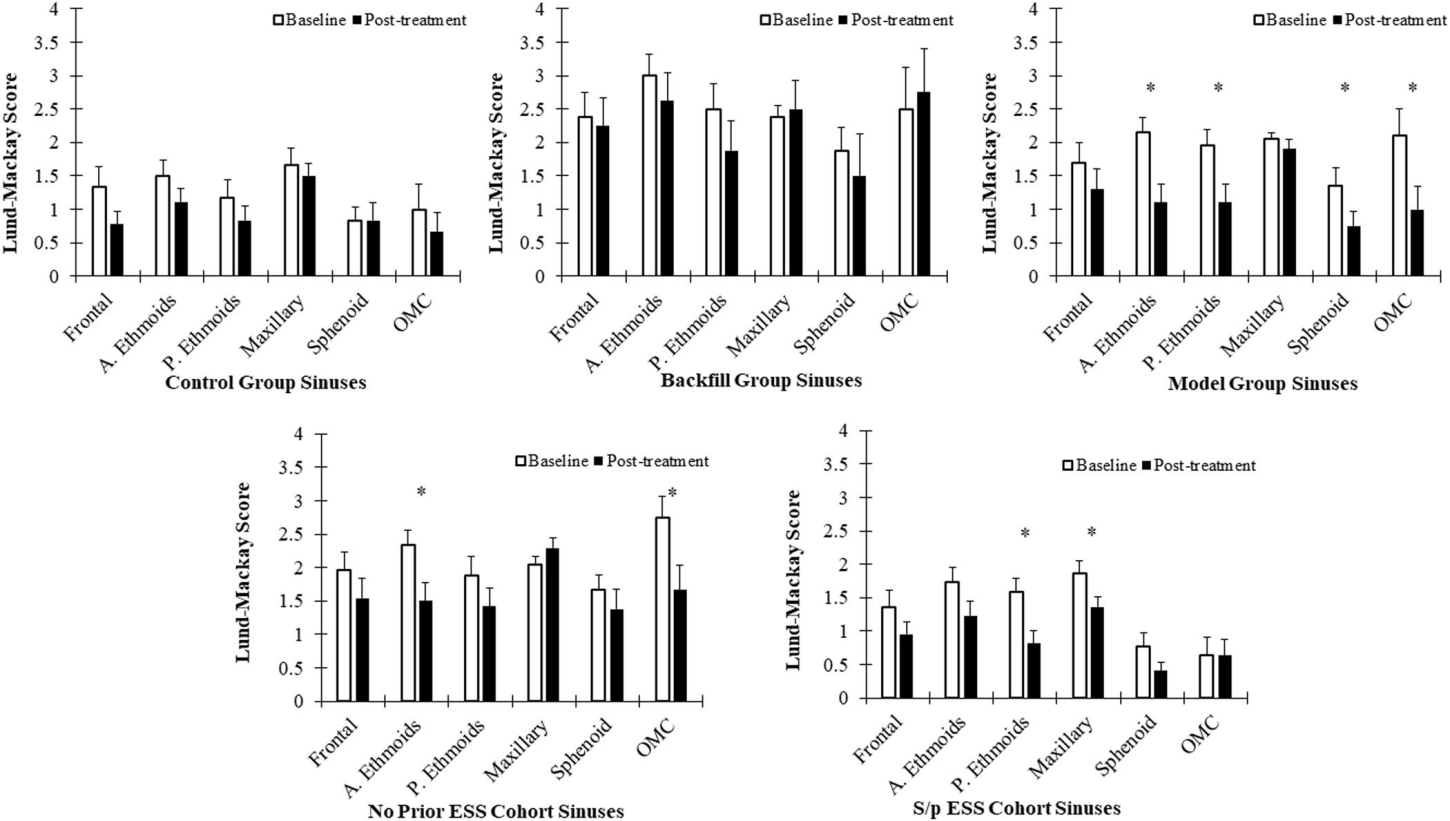
Plots of sinus‐specific LM score improvement after treatment among the randomized groups, as well as among cohorts with or without prior surgery. A. Ethmoids, anterior ethmoids; ESS, endoscopic sinus surgery; LM, Lund‐Mackay; OMC, ostiomeatal complex; P. Ethmoids, posterior ethmoids. *indicates statistical significance of *p* < 0.05.

SNOT‐22 score significantly improved for each group posttreatment, as did the NOSE score and VAS of nasal congestion (Figure 2, Table 2). The MG showed greater improvement in all patient‐reported outcome measures (PROMs) than the other 2 groups; however, the interaction between timepoints and the treatment group was not statistically significant, meaning the improvement in MG was not significantly better than the other groups. Significant posttreatment improvement in objective LM scoring was only observed in the MG (−3.73, 95% confidence interval = −5.71, −1.75; *P* < .001), but not in BG and CG groups (*P* = .400 and .405, respectively), even though MG had the least number of patients with prior sinus surgery (n = 3/11). All baseline variables were not statistically different, except that the LM score was significantly higher in the BG than the CG (mean 13.7 vs 7.1, *P* = .006). Sinus‐specific LM scoring also showed that MG patients had a significant reduction in ostiomeatal complex (OMC) occlusion and opacification in the ethmoid and sphenoid sinuses (Figure 3). The CG and BG had no significant improvement in any of the sinuses.

**Table 2:** Linear Mixed Model Analysis

Measure	Treatment group or surgical cohort	Difference (post‐pre)	95% CI	*P* value	Interaction *P* value
LM	Backfill	−1.31	−4.48, 1.85	.400	.128
LM	Control	−0.88	−3.04, 1.27	.405	.128
LM	Model	−3.73	−5.71, −1.75	<.001	.128
LM	No prior surgery	−2.27	−4.18, −0.35	.023	.940
LM	Prior surgery	−2.16	−4.24, −0.08	.042	.940
NOSE	Backfill	−26.36	−39.74, −12.99	<.001	.660
NOSE	Control	−26.43	−38.28, −14.58	<.001	.660
NOSE	Model	−33.64	−47.01, −20.26	<.001	.660
NOSE	No prior surgery	−32.65	−43.19, −22.10	<.001	.292
NOSE	Prior surgery	−25.00	−34.97, −15.03	<.001	.292
SNOT‐22	Backfill	−13.64	−24.51, −2.76	.016	.525
SNOT‐22	Control	−17.32	−26.96, −7.68	<.001	.525
SNOT‐22	Model	−22.27	−33.15, −11.40	<.001	.525
SNOT‐22	No prior surgery	−17.06	−25.83, −8.28	<.001	.837
SNOT‐22	Prior surgery	−18.29	−26.59, −9.99	<.001	.837
VAS	Backfill	−1.37	−2.72, −0.03	.046	.896
VAS	Control	−1.73	−2.92, −0.53	.006	.896
VAS	Model	−1.76	−3.11, −0.42	.012	.896
VAS	No prior surgery	−1.49	−2.56, −0.42	.008	.711
VAS	Prior surgery	−1.76	−2.77, −0.75	.001	.711

A linear mixed model with random intercept was used to compare within the treatment group and between treatment groups outcomes while adjusting for prior surgical status, age, gender, medication history (antibiotic, antihistamine, and steroid use), and polyp status. The interaction between the group and treatment was also examined. Additionally, a subgroup analysis stratifying patients based on prior surgical history while adjusting for the treatment group, age, gender, medication history (antibiotic, antihistamine, and steroid use), and polyp status was performed. The interaction between prior surgical status and treatment was also examined. Overall, the MG showed greater improvement in all measures though it was not statistically significant as compared to the CG or BG (all interaction *P* > .05).

Abbreviations: BG, backfill group; CG, control group; CI, confidence interval; LM, Lund‐Mackay staging score; MG, model group; NOSE, Nasal Obstruction Symptom Evaluation scale; SNOT‐22: Sinonasal Outcome Test; VAS, visual analog scale of nasal obstruction.

Among the 36 patients, 19 patients had prior functional endoscopic sinus surgery (s/p ESS) (9 CG, 7 BG, 3 MG), ranging from 6 months to 34 years ago (median 6 years), while the other 17 subjects had no prior ESS history. Patients were further stratified based on prior surgical status, showing that both the surgical and non‐surgical cohorts had significantly improved PROMs and LM scores posttreatment (Figure 2, Table 2) with the improvement between the cohorts not being significantly different. However, when comparing the surgical and nonsurgical cohorts in sinus‐specific LM scoring, prior surgical patients had significant improvement in maxillary and posterior ethmoid sinus opacification as well as less occluded baseline OMCs. The patients without prior surgery had significant improvement in anterior ethmoid and OMC opacification (Figure 3). Results remained consistent after controlling for antibiotic, antihistamine, and steroid use in the linear mixed model.

**Figure 4:**
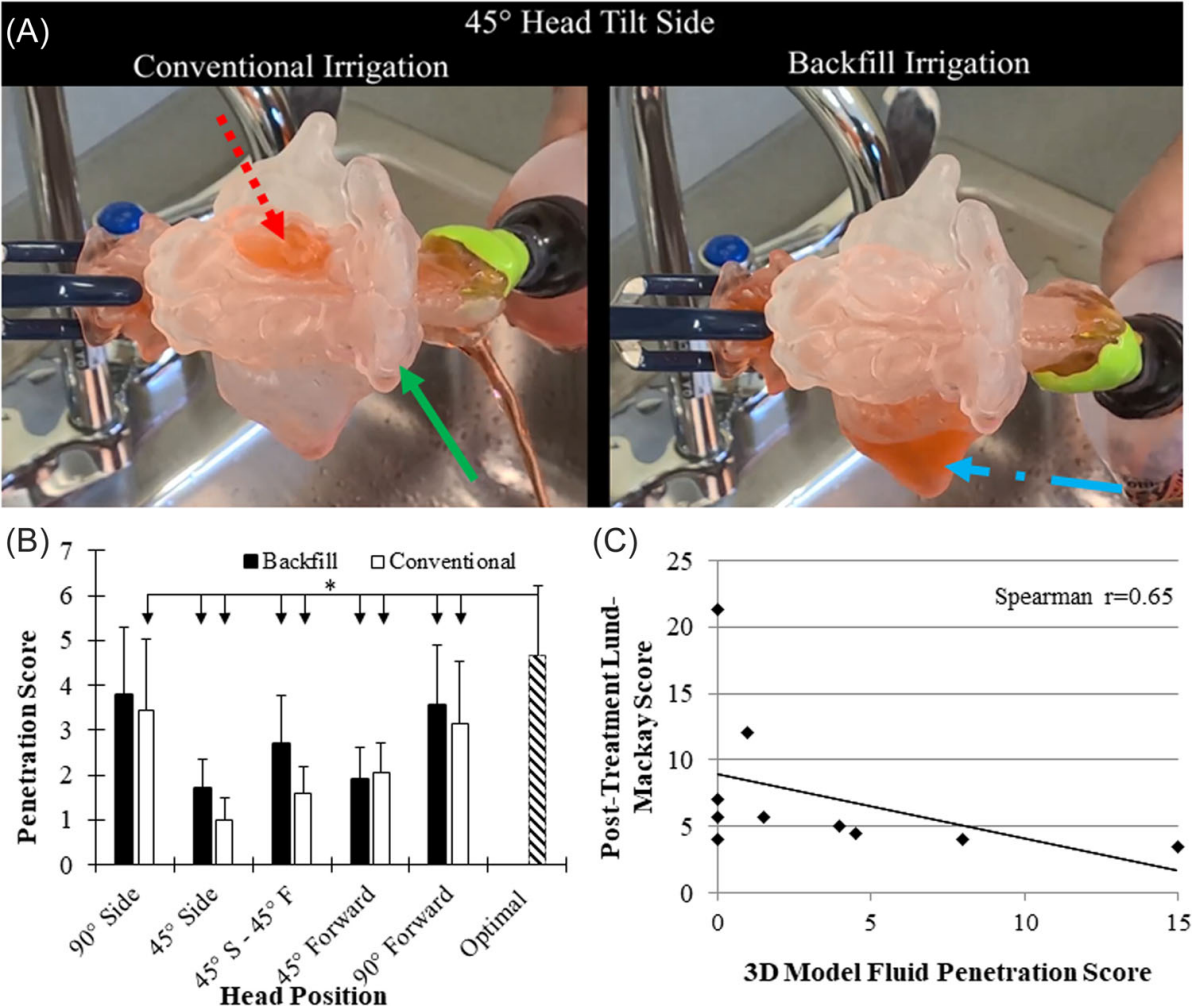
(A) Visualization of fluid penetration for 1 patient in the conventional and backfill 45° tilt to the side position. The model is photographed in the axial view, with fluid entering and exiting the nostrils on the right side of the image. Dashed red arrow: full irrigant filling of the left ethmoid sinus, earning a penetration grade of 3. Solid green arrow: no penetration of the right frontal sinus, earning a penetration grade of 0. Dash‐dot blue arrow: partial filling of the right maxillary sinus, earning a penetration grade of 2. (B) Averaged penetration score of each head position for all patients as well as the averaged penetration score in the final optimized head position and irrigation paradigm (eg, backfill vs conventional). (C) The final optimal penetration score significantly correlates to posttreatment LM score (Spearman's *r* = 0.65, *P* < .05). LM, Lund‐Mackay.

The optimal irrigation strategy as determined by the 3D printed model varied greatly among the MG: n = 5 with 90° side tilt backfill, n = 5 with 90° forward tilt conventional, n = 2 with 45° side tilt backfill, n = 2 with 45° side tilt conventional, n = 1 with 45° forward tilt and 45° with side tilt backfill, and n = 1 with 45° forward tilt. The backfill position was prescribed to subjects whose models did not allow for any fluid penetration to the sinuses in any positions tried (n = 2 bilateral and n = 2, 1‐sided). Eight MG subjects maintained their prescribed head position and 3 changed to a different position after their own at‐home practice with the model. Among the MG, if every patient is assigned to their final optimal position, the resulted sinus penetration scores were significantly higher than if any one of the other head positions was assigned to all patients (*P* < .05), except for the 90° side tilt backfill, over which a notable but not significant difference was observed (Figure 4). The resultant sinus penetration scores of the actual position used by the patients correlated strongly to the final LM outcome (Spearman's *r* = 0.65, *P* < .05). Among patients who completed the trial, there was no difference in patient‐reported compliance between the groups (CG: 6.8 days per week; BG: 6.3; MG: 6.6, *P* > .05).

## Discussion

In this study, we report the first randomized clinical trial using 3D‐printed sinonasal models to determine whether the development of a personalized irrigation method is more effective in treating CRS than the traditional generalized methods of irrigation while taking into account the effects of prior surgical history. Intuitively, the standard position (CG) allows fluid flow through the upper nostril, driven by gravity, to fill the paranasal cavity and exit out the lower nostril. However, there has been little objective evidence to confirm that this is the optimal technique for nasal irrigation. Our previous 3D printing study has shown that pushing the irrigant against gravity through the lower nostril can allow the irrigant to pool around the ostium and improve penetration into the maxillary sinus, which we call the backfill technique.^20^, ^24^ In addition to these 2 generalized instructions, we used 3D‐printed sinonasal models to determine the optimal head position for each patient assigned to the MG.^19^, ^20^

Patients in all groups showed significant improvement in PROMs (NOSE, SNOT‐22, and VAS) with the MG showing greater improvement than the other 2 groups, although this was not statistically significant. Objective LM scores showed statistically significant improvement only in the MG, but not the CG or BG. Clinically, the backfill technique does not seem to objectively reduce sinus inflammation compared to other head positions, which contradicts the fact that some of the prescribed optimal positions for the MG closely resembled the backfill position. While future large‐scale studies are warranted for further investigation of the backfill position, this finding does support the notion that a one‐position‐fits‐all approach may not result in optimal clinical outcomes.

Oftentimes, ESS is used to allow for more effective topical medication delivery. A previous clinical trial of sinus irrigation only included postoperative patients and argued that unoperated sinuses receive ineffective amounts of topical medication.^5^ Here, we showed both patients with or without prior surgical history had similar degrees of improvement in subjective PROM scores as well as LM scores, although they do have their unique differences. Patients with prior surgical history had significantly lower baseline objective LM scores and less occluded baseline OMCs. This is clinically expected since the key target of ESS often involves the OMC. These less occluded OMCs may have allowed for more medication delivery to the maxillary and ethmoid sinus which resulted in a significant reduction in opacification in these sinuses. Conversely, patients without prior surgery had significantly occluded baseline OMCs, but with significant improvement after irrigation treatment. It is interesting that this improved OMC did not necessarily translate to improvement in the maxillary sinus, which actually increased inflammation. We speculate that 2 months of irrigation may not be adequate to allow for OMC opening and sufficient time for drug delivery to the maxillary sinus. A longer treatment course might be necessary to observe a clinical effect in patients without prior surgery and allow for a gradually cleared OMC to facilitate irrigation delivery to paranasal sinuses, and this may be tested in future clinical trials.

Figure 4 shows a significant correlation between optimal position sinus penetration score and posttreatment LM score in the MG. Of note, a subset of MG subjects (n = 2) had a total penetration score < 2 for all sinuses combined even at the optimal irrigation positions tested, and had poor clinical outcomes (LM > 10). While both patients did not have prior surgery, surgical status may not be the only crucial predictive factor, as 3 of the 5 patients with high penetration scores and good clinical outcomes also had no prior surgery, indicating innate anatomical variation can also be an important factor. In the future, individual 3D printed models have the potential to screen for the subset of patients with poor sinus penetration even with the optimal irrigation paradigm, where perhaps immediate surgery would be a better option for them than a likely ineffective irrigation treatment course.

While there was no difference in daily compliance between the patients who remained in the study, CG patients had a higher voluntary drop‐out rate (7/23 = 30%) than the MG (3/17 = 17.6%) and BG (1/23 = 4.3%), which is also an indicator of compliance. The MG received more engagement through teaching and was encouraged to practice with their 3D model at home, which may have contributed to a lower voluntary dropout rate in this group. The subjective outcome may be biased since only patients with subjective improvement continued their treatment and stayed in the trial. It is also reasonable to argue that “compliance” may not equal proper technique. Nasal irrigation can be a subjectively uncomfortable experience for the first time, and it can take time and practice before proper head positioning is achieved. Allowing patients to choose their own method of medication administration and providing teaching based on the patient's individual needs is all part of providing patient‐centered care.^26^, ^27^

A key limitation of this study is that only 56% of the enrolled cohort completed the study, which reduced power and prevented some trending variables from reaching statistical significance. Even though the linear mixed model statistical test can account for confounding factors, including missing data and differing baseline scores, it is difficult to make concrete conclusions when a significant difference is present in the baseline LM score between the BG and CG. This may have been due to our difficulty in accessing CT scans from outside referral clinics, resulting in a disparity of complete LM scores between groups (11 in CG, 5 in BG, and 11 in the MG). However, it is still reasonable to say that a high baseline score does not necessarily result in greater LM improvement, as the BG had the highest average baseline LM score yet also had the lowest improvement. This reaffirms our decision to use a linear mixed model rather than a simple descriptive comparison method (ie, Student's *t* test). Nevertheless, replication of this pilot study in a larger sample size could potentially verify our novel and preliminary findings with greater statistical power.

## Conclusion

A one‐size‐fits‐all irrigation strategy may not exist. However, using 3D printing to create a personalized, optimal head position for mometasone irrigations can result in objectively less inflammation in patients with CRS as compared to traditional irrigation paradigms. While patients with prior ESS began mometasone treatment with objectively lower inflammation, these patients experienced similar baseline symptom burden, similar symptom reduction, and similar total inflammation reduction as compared to patients without prior ESS.

## Author Contributions

**Zachary T. Root**, manuscript author, lead study coordinator, data analysis; **Thomas J. Lepley**, obtained Institutional Review Board approval, assistant study coordinator; **Kanghyun Kim**, 3‐dimensional (3D) model printing and experimentation; **Aspen R. Schneller**, computational 3D modeling; **Songzhu Zhao**, statistical analysis; **Raymond Wen**, model experimentation; **Veronica L. Formanek**, assisted with patient follow‐up; **Sarah M. Sussman**, Lund‐Mackay scoring; **Joseph S. Lee**, Lund‐Mackay scoring; **Ahmad Odeh**, revised the limitation section; **Lai Wei,** statistical analysis; **Kathleen M. Kelly**, physician, Lund‐Mackay scoring, manuscript revision; **Bradley A. Otto**, physician, manuscript revision; **Kai Zhao**, principal investigator.

## Disclosures

### Competing interests

Kai Zhao is a consultant to Diceros Therapeutics Inc.

### Funding source

NIH R01DC020302 to Kai Zhao.
